# Editorial Comment: The Basic Physics of Waves, Soundwaves, and Shockwaves for Erectile Dysfunction

**DOI:** 10.1590/S1677-5538.IBJU.2020.05.08

**Published:** 2020-07-31

**Authors:** Valter Javaroni

**Affiliations:** 1 Hospital Federal do Andaraí Rio de Janeiro Departamento de Andrologia Rio de Janeiro RJ Brasil Departamento de Andrologia, Hospital Federal do Andaraí Rio de Janeiro, Rio de Janeiro, RJ, Brasil

Katz JE ^1^, Clavijo RI ^2^, Rizk P ^1^, Ramasamy R ^3^

## COMMENT

Dr. Jonathan Elliott Katz and cols., taking into consideration that Li-ESWT is not U.S. Food and Drug Administration approved and is considered investigational in the United States and available to patients under clinical trial protocols, made a nice review on the physic aspects of shockwaves. And drive attention to the aspect that each device should use the protocol approved for it.

In 2010, Vardi et al. (1) published their first paper, which introduced the use of low intensity extracorporeal shock wave therapy (LI-ESWT) for ED. Since them, many articles have been published communicating safety and efficacy of Li-ESWT in ED patients (2). The results of studies with a small number of patients and a short observation period were encouraging, but we live with the diversity of devices and the lack of clear protocols, mainly in special populations.

In a recent report, Dr. Fojecki and cols. (3) presented 6- and 12-month data from a randomized, sham-controlled trial on LI-ESWT for ED and did not find any clinically significant effect between two different protocols.

According to another recent publication on Journal of Sexual Medicine (4): “*These studies provide preliminary insights, but no definitive answers, and many questions remain unanswered regarding the mechanism of action, as well as the ideal treatment protocol”.*

However, there is a need to define which subgroup of ED population is best suited and the most appropriated LI-ESWT treatment protocol including template, modality of shockwaves energy, emission frequency, and total energy delivery.

So, we still do require better design studies with clear inclusion and exclusion criteria using validated tools to access erectile function improvement in long follow up periods. Ideally we should do it before treating our patients.
